# Growth mindset and adolescent exercise persistence: a moderated serial mediation model through self-efficacy and intrinsic motivation

**DOI:** 10.3389/fpsyg.2026.1860747

**Published:** 2026-09-17

**Authors:** Xiaoliang Huo, Ming Hui

**Affiliations:** 1 School of Physical Education, Tibet University for Nationalities, Xianyang, Shaanxi, China; 2 Sports College, Chongqing University of Posts and Telecommunications, Nan’an, Chongqing, China

**Keywords:** Chinese adolescents, exercise persistence, exercise self-efficacy, growth mindset, intrinsic motivation, perceived exercise climate

## Abstract

Adolescence is the period in which physical-activity habits either take root or quietly unravel, and in China crowded timetables and examination pressure make the second outcome unusually common. What separates the adolescents who keep exercising from those who drift away is therefore a question with real public-health stakes, and beliefs about whether athletic ability can be developed offer one promising but under-tested answer. Drawing on social cognitive theory and self-determination theory, we specified a moderated serial mediation model and examined it in 860 adolescents aged 12 to 18, sampled from eastern, central, and western China. Growth mindset, exercise self-efficacy, intrinsic motivation, perceived exercise climate, and baseline persistence were assessed at Time 1; exercise persistence was reassessed 1 month later and served as the outcome, with baseline persistence controlled. The analytic sequence combined confirmatory factor analysis, tests of measurement invariance across gender and school level, structural equation modeling with cluster-robust standard errors, bias-corrected bootstrapping with 5,000 resamples, and an unconstrained product-indicator test of latent moderation. Growth mindset was associated with exercise persistence both directly (*β* = 0.14) and indirectly through exercise self-efficacy (*β* = 0.110), through intrinsic motivation (*β* = 0.082), and through the sequential chain running from self-efficacy to intrinsic motivation (*β* = 0.053); the three indirect paths together accounted for 63.6% of the total association. Perceived exercise climate moderated the first stage, so that the mindset-to-efficacy link was close to twice as strong under mastery-oriented perceptions (index of moderated mediation = 0.032, 95% CI [0.011, 0.058]). The pattern survived the addition of demographic covariates, the control of baseline persistence, and adjustment for school-level clustering, although a design of this kind licenses statements about association rather than about causal mechanism. Practically, the findings suggest that work on adolescents’ beliefs and work on the climates they train in belong together rather than apart.

## 1 Introduction

Whether adolescents believe their athletic abilities can be built through effort, or instead regard them as fixed endowments, shapes how they approach physical activity over time. The distinction traces back to Dweck and Yeager (2019) implicit theories of intelligence and has since generated a substantial body of work in educational and organizational psychology. Its migration into sport and exercise settings has been slower and considerably less systematic, particularly among younger participants whose long-term activity habits are still forming. Our own interest in the question began with a plain observation: Chinese secondary schools invest heavily in physical education, yet participation still falls away sharply across the adolescent years. If beliefs about the malleability of athletic ability help explain who keeps going, they would be an unusually inexpensive lever to pull. The present study accordingly asks how growth mindset relates to exercise persistence among Chinese adolescents, and traces the mediating and moderating pathways that might connect a belief to a behavior.

Dweck and Leggett (1988) original framework proposed that individuals holding an incremental (growth) view of ability tend to adopt mastery-oriented goals, embrace challenge, and persist after setbacks, whereas those endorsing an entity (fixed) view gravitate toward performance goals and withdraw from difficulty. Subsequent work carried these ideas beyond academic achievement into creativity, leadership, and health behavior (Burnette et al., 2013). The last 5 years, however, have complicated the picture rather than confirming it. A large national experiment found that a brief mindset intervention raised grades chiefly among lower-achieving students, and only where peer norms supported the message (Yeager et al., 2019); two meta-analyses published in the same volume reached sharply different verdicts about whether such interventions work at all once design quality and publication bias are taken into account (Burnette et al., 2023; Macnamara and Burgoyne, 2023). Read together, this literature suggests that mindset effects are neither uniform nor large, and that they depend heavily on who holds the belief and on what the surrounding environment does with it. Evidence from sport and exercise is similarly uneven. Work with elite track-and-field athletes has traced both the antecedents and the consequences of implicit beliefs about ability (Jowett and Spray, 2013), and early cross-sectional research among adult exercisers linked incremental beliefs to intrinsic motivation and sustained engagement (Kasimatis et al., 1996), and a longitudinal study of collegiate athletes reported that preseason growth mindset predicted greater training effort 6 months later, independent of initial performance (Stenling et al., 2022). What these studies have rarely done is model the intervening processes explicitly, and adolescent samples remain scarce.

One plausible pathway runs through self-efficacy. Social cognitive theory holds that efficacy beliefs regulate effort allocation, emotional responses, and perseverance in the face of obstacles (Bandura, 1997). An adolescent who treats athletic skill as improvable may read early struggle as part of a learning curve rather than as evidence of permanent inadequacy, and so preserve confidence in her capacity to succeed. Academic research supports this reasoning: growth mindset has been linked to higher self-efficacy, which in turn predicted greater persistence (Cherewick et al., 2023), and a recent study of Chinese school students found that grit and academic self-efficacy transmitted the effect of growth mindset in serial sequence, albeit onto a psychological rather than a behavioral outcome (Meng et al., 2025). Whether an analogous chain operates in exercise settings has received little direct testing. A second pathway involves intrinsic motivation. Self-determination theory argues that behavior endures when it satisfies the basic needs for autonomy, competence, and relatedness, thereby fuelling autonomous forms of regulation (Ryan and Deci, 2020). Meta-analytic syntheses in physical education and in the wider health domain confirm that autonomous motivation ranks among the more dependable correlates of sustained engagement (Vasconcellos et al., 2020; Ntoumanis et al., 2021), and growth-minded individuals, by attending to personal improvement rather than normative comparison, may experience greater need satisfaction during physical activity (Wang and Biddle, 2007). Each link in the sequence from mindset through efficacy to motivation and finally to persistence has been documented on its own; to our knowledge, the links have not yet been estimated together as a single chain in the exercise domain.

Beyond individual cognition, the social environment plausibly shapes how a mindset translates into action. Perceived exercise climate, meaning the degree to which an adolescent senses that coaches, peers, and parents reward effort and improvement rather than innate talent, may amplify or dampen the mindset-persistence link (Duda, 2001). A mastery-oriented climate supplies exactly the external evidence on which incremental beliefs rest, namely that effort yields progress, whereas a performance-oriented climate can quietly contradict that premise even for growth-minded youth (Ntoumanis and Biddle, 1999). Climate is also the contextual variable most directly commensurable with mindset, since both concern the meaning attached to effort; that correspondence, rather than any claim that other contextual influences are unimportant, is our reason for singling it out. Despite the theoretical rationale, its moderating role on the mindset-to-persistence pathway has seldom been examined empirically, and almost never among adolescents outside Western settings.

Several methodological limitations further constrain what the current literature can support. Most instruments assessing growth mindset in physical activity were developed for adults and validated in Western samples, so their psychometric adequacy for Chinese adolescents remains uncertain, and few studies report whether their measures function equivalently across gender or school level (Li et al., 2021). Many designs have been simply bivariate, testing direct associations between mindset and behavior without the mediators and moderators that theory would predict (Burnette et al., 2023). School-based samples are typically clustered within classes and schools, yet that structure is rarely reflected in the standard errors. Sample sizes are seldom justified against the small indirect effects such models are meant to detect. The adolescent population is itself under-represented, since much of the sport mindset literature draws on collegiate or professional athletes, leaving open whether similar processes operate during the years when activity habits crystallise or erode. Chinese adolescents, finally, face particular pressures, including heavy academic workloads, exam-driven scheduling, and cultural attitudes that may privilege scholastic over athletic achievement, which make exercise persistence both especially valuable for health and especially difficult to sustain.

This study therefore pursues three objectives. First, we test whether growth mindset in the exercise domain is positively associated with adolescent exercise persistence. Second, we construct and evaluate a serial mediation model in which exercise self-efficacy and intrinsic motivation sequentially transmit that association. Third, we examine whether perceived exercise climate moderates the first stage of the indirect pathway, specifically the link from growth mindset to exercise self-efficacy. Because a serial model of this kind places demands on both design and statistical power, we determined the target sample *a priori* through Monte Carlo simulation, measured persistence again after 1 month so that the outcome could be modeled with baseline persistence controlled, and subjected the estimates to a series of robustness checks. By joining propositions from social cognitive theory and self-determination theory within a single moderated mediation framework, the study offers a more complete account of how mindset beliefs operate in context than bivariate designs allow (Preacher et al., 2007). The findings also carry practical weight: if mindset is related to persistence mainly through efficacy and motivation, then programmes aimed at adherence should address belief systems alongside skill instruction, and if climate perception conditions that process, environmental restructuring becomes an additional lever.

The contribution of this work is threefold. Conceptually, it bridges implicit theories research and motivational science by specifying, and then estimating, the chain through which a general belief orientation may convert into a specific behavioral pattern. Methodologically, it adapts and evaluates measurement tools in a Chinese adolescent sample, reporting invariance evidence and cluster-adjusted estimates that cross-cultural work in exercise psychology has often omitted. Empirically, it provides what we believe to be among the first tests of a multi-path moderated mediation model linking growth mindset to exercise persistence among non-adult, non-Western participants, a population whose activity trajectories carry long-term public-health consequences. We state that claim comparatively rather than absolutely, since the relevant literature is scattered across education, sport science, and public health, and absence of evidence in one of them is weak grounds for a claim of priority.

## 2 Theoretical foundations and research hypotheses

### 2.1 Theoretical framework of growth mindset and its extension to sport contexts

Dweck (1999) implicit theories framework distinguishes two lay beliefs about human attributes: an entity theory, which treats ability as a stable, innate quantity, and an incremental theory, which frames ability as a quality that expands through practice and strategic effort. The distinction is not merely attitudinal—it reorganizes how individuals interpret failure, set goals, and regulate motivation. Entity theorists tend to read poor performance as diagnostic of low capacity; incremental theorists treat the same outcome as informative feedback (Yeager and Dweck, 2020).

Originally validated in academic achievement research, the framework migrated into sport psychology through work on athletes’ conceptions of physical talent. Biddle and colleagues were among the first to show that implicit beliefs about athletic ability predicted goal orientation and affective responses to competition in youth sport samples (Biddle et al., 2003). Later adaptations refined that transfer by arguing that the domain specificity of mindset matters: a student may hold an incremental view of intelligence while endorsing an entity view of coordination or speed (Spray et al., 2006). The observation prompted researchers to build sport-specific measures rather than repurpose academic scales. The Conceptions of the Nature of Athletic Ability Questionnaire represented an early effort, although its factor structure proved unstable across age groups (Sarrazin et al., 1996). More recent instruments anchor their item stems in concrete physical capacities, for example one’s running speed, in order to capture beliefs tied to specific competencies (Slater et al., 2012). The methodological debate that has since developed around mindset effects makes this precision more, not less, important, because weak measurement is one of the mechanisms through which inflated effects enter a literature (Macnamara and Burgoyne, 2023).

For the present study, we define growth mindset in the exercise context as the degree to which an individual endorses the belief that her sport-related competencies—strength, endurance, technique—can be improved through sustained effort and deliberate training. This operationalization aligns with Bandura (2001) social cognitive account, wherein personal beliefs about capability interact with outcome expectations and environmental supports to regulate behavior. From this vantage point, a growth-oriented exerciser does not simply “try harder”; she constructs higher efficacy expectations, selects more challenging tasks, and persists longer when progress stalls—behavioral signatures that map directly onto the persistence construct we aim to predict (Bandura, 2004).

### 2.2 Predictors and explanatory models of exercise persistence behavior

Exercise persistence refers to an individual’s continued participation in physical activity over time despite barriers such as fatigue, competing demands, and motivational fluctuations—a construct typically assessed along dimensions of behavioral frequency, duration of adherence, and resistance to dropout (Sallis et al., 2000). Several theoretical traditions have attempted to account for why some adolescents maintain regular exercise while others abandon it. The theory of planned behavior locates persistence in the alignment of attitudes, subjective norms, and perceived behavioral control (Ajzen, 1991). Self-determination theory shifts attention to motivational quality, predicting that autonomously regulated behavior endures longer than behavior driven by external contingencies (Ryan and Deci, 2020). Social-ecological models, by contrast, distribute causal weight across individual, interpersonal, and institutional layers, treating persistence as a product of nested environmental influences rather than purely intrapersonal processes (Sallis et al., 2008).

What cuts across these frameworks is a recurring emphasis on two proximal determinants: self-efficacy and intrinsic motivation. Meta-analytic evidence places exercise self-efficacy, meaning confidence in one’s ability to carry out a planned physical activity routine, among the strongest individual-level predictors of sustained engagement in adolescent samples (Dishman et al., 2004). Intrinsic motivation captures the experiential pull of the activity itself; adolescents who find exercise enjoyable or personally meaningful are far more likely to continue than those who exercise under parental or institutional pressure (Crocker et al., 2006), and large syntheses in physical education and health behavior report the same ordering of motivational quality (Vasconcellos et al., 2020; Ntoumanis et al., 2021). The two constructs are not independent. Efficacy beliefs feed forward into motivational quality, because feeling capable of performing an activity tends to satisfy the need for competence, which in turn nourishes autonomous motivation (Ryan and Deci, 2017).

Where does growth mindset fit within this predictive architecture? We treat it as a distal cognitive antecedent, a belief orientation that sets initial conditions for the more proximal motivational variables. An adolescent convinced that athletic competence is expandable should, on that basis alone, appraise her exercise capacity more favourably and experience physical activity as a domain of personal growth rather than obligatory performance. The resulting ordering, from mindset to efficacy to motivation to persistence, positions growth mindset upstream of the established proximal predictors rather than as a competitor for the same explanatory variance (Yeager and Dweck, 2012). We use the language of ordering deliberately: the sequence is a theoretical proposition about how the constructs are arranged, and the present design tests its statistical implications rather than its causal truth.

### 2.3 Research hypotheses and theoretical model construction

Drawing on the theoretical reasoning developed above, we advance five hypotheses. The first concerns the direct path: growth mindset should positively predict exercise persistence even before mediators are introduced, because incremental beliefs orient adolescents toward mastery goals that sustain effort (Dweck and Leggett, 1988). The direct effect can be expressed as Equation 1 in standard regression notation (following Hayes (2022) process modeling conventions):

$$Y=c^{\prime }X+e_{1}$$ (1)

where Y denotes exercise persistence, X denotes growth mindset, $c^{\prime }$ represents the direct effect coefficient, and $e_{1}$ is the residual term.

*H1*: Growth mindset positively predicts adolescent exercise persistence behavior.

Two simple mediation hypotheses follow. If growth mindset elevates exercise self-efficacy ( $M_{1}$ ), and self-efficacy in turn predicts persistence, then $M_{1}$ transmits part of the mindset effect (Bandura, 2001). Parallel logic applies to intrinsic motivation ( $M_{2}$ ): growth-oriented adolescents should gravitate toward autonomous engagement, which then sustains behavior (Vallerand, 1997). The single-mediator path is formalized as Equation 2:

$$M_{1}=a_{1}X+e_{2},Y=c^{\prime }X+b_{1}M_{1}+e_{3}$$ (2)

*H2*: Exercise self-efficacy mediates the relationship between growth mindset and exercise persistence.

*H3*: Intrinsic motivation mediates the relationship between growth mindset and exercise persistence.

The chain mediation hypothesis, our core theoretical proposition, integrates both mediators sequentially. Three considerations place self-efficacy ahead of intrinsic motivation rather than beside or behind it. First, self-determination theory treats competence satisfaction as an input to autonomous motivation, and an efficacy appraisal is precisely the cognitive judgement through which perceived competence is registered (Ryan and Deci, 2017). Second, Bandura (1997) account assigns efficacy beliefs a regulatory role that precedes interest and valuation, since people rarely develop enduring interest in activities they expect to perform badly. Third, the empirical ordering has been observed elsewhere: growth mindset predicted downstream outcomes through efficacy-type mediators in Chinese school samples (Meng et al., 2025), and efficacy has predicted subsequent autonomous motivation more consistently than the reverse in physical education research (Vasconcellos et al., 2020). We therefore expect growth mindset to raise self-efficacy first, which then strengthens intrinsic motivation, which finally supports persistence. The sequential model takes the form (Equation 3):

$$M_{2}=a_{2}X+d_{1}M_{1}+e_{4},Y=c^{\prime }X+b_{1}M_{1}+b_{2}M_{2}+e_{5}$$ (3)

*H4*: Exercise self-efficacy and intrinsic motivation sequentially mediate the effect of growth mindset on exercise persistence (chain mediation).

Two qualifications belong with this hypothesis. The reverse ordering is not implausible: adolescents who already find an activity absorbing accumulate practice and thus experience, which can raise efficacy in turn, and reciprocal influence over time is entirely compatible with both theories. Data collected on a single occasion cannot adjudicate between these arrangements, because models that differ only in the direction of the mediator-to-mediator path frequently fit observed covariances almost equally well (Maxwell and Cole, 2007). We therefore estimate the alternative ordering as a competing model and report the comparison rather than assuming that the hypothesized sequence is the only one the data support.

Finally, we expect the social environment to condition the first link in this chain. Adolescents who perceive a mastery-oriented exercise climate should convert growth beliefs into self-efficacy more readily than those immersed in performance-oriented settings, because environmental cues confirming that effort yields improvement reinforce the very premise on which incremental beliefs rest (Ames, 1992). Perceived climate is not the only contextual factor that might play this role; parental sport socialisation and peer exercise norms are obvious rivals. We privilege climate here for three reasons: it shares with mindset the same underlying currency, namely the meaning assigned to effort; it has a mature measurement tradition within achievement goal theory; and, unlike family background, it is directly modifiable by teachers and coaches, which matters if the results are to inform practice.

*H5*: Perceived exercise climate moderates the path from growth mindset to exercise self-efficacy, such that this relationship is stronger under high mastery climate perceptions.

Together, *H1* through *H5* compose a moderated serial mediation model, an integrated architecture that nests individual cognition within its social context and traces the full transmission chain from a distal belief to a proximal behavior (Preacher et al., 2010). Throughout, the hypotheses should be read as predictions about patterns of association implied by that architecture. Establishing the causal claims they are derived from would require experimental manipulation or repeated measurement of every construct, neither of which the present design provides.

## 3 Methods and data collection

### 3.1 Participants and research design

A stratified random sampling procedure was used to recruit adolescents from three geographic tiers in China, namely eastern, central, and western provinces, so as to span socioeconomic and regional variation (Cochran, 1977). Within each tier we randomly selected two cities, and within each city one junior high school and one senior high school, giving 12 participating schools. School principals were approached through the provincial education bureaus; all 12 schools contacted agreed to take part. Intact class groups were then randomly sampled within each school, yielding 30 classes and an initial pool of 912 students. Students were eligible if they were enrolled in regular physical education, were aged between 12 and 18, and provided assent alongside guardian consent; those with a medical exemption from physical education, or with an injury preventing exercise during the study window, were excluded. Fifty-two students were subsequently removed because of incomplete responses or failed attention-check items, leaving a final analytic sample of 860 (94.3% of the initial pool). Socioeconomic background was recorded through parental education and family residence: 41.9% of participants had at least one parent with post-secondary education, and 63.5% lived in urban districts. Because students were nested within classes and schools, intraclass correlations were computed for all study variables and the structural model was estimated with standard errors adjusted for clustering at the school level, an approach that preserves the single-level specification while protecting inference against the dependency that intact-class sampling introduces (McNeish et al., 2017).

The sample size was fixed in advance rather than by convenience. Using Monte Carlo simulation for indirect effects with 5,000 replications per condition, and seeding the simulation with standardized path values drawn from prior work on mindset, efficacy, and motivation, we estimated that 347 cases would be needed for 0.80 power to detect a serial indirect effect of about 0.05, and 571 cases for the index of moderated mediation (Schoemann et al., 2017). Anticipating a design effect close to two from the clustered sampling frame, we doubled the larger of these figures and rounded upward, then inflated the target by roughly 6% to absorb attrition between waves, which produced a recruitment goal of about 900 students. The realized sample of 860 exceeds the simulated requirement for every path in the model, although, as the robustness analyses in Section 4.3 make clear, the moderated mediation index remains the least well-powered quantity we estimate.

Table 1 summarizes the demographic profile of this sample. The gender split was roughly balanced (males: 53.1%; females: 46.9%), with ages ranging from 12 to 18 years (*M* = 14.82, SD = 1.76). Participants were distributed across junior high (grades 7–9; 51.6%) and senior high (grades 10–12; 48.4%). The most common sport types were ball sports (38.7%), track and field (24.5%), swimming (15.3%), and other activities (21.5%). Exercise experience varied: 32.8% reported fewer than 2 years of regular participation, 41.2% reported 2 to 5 years, and 26.0% exceeded 5 years.

**Table 1:** Demographic characteristics of participants (*N* = 860).

Variable	Category	*n*	%	M (SD)
Gender	Male	457	53.1	—
	Female	403	46.9	—
Age (years)	12–18	860	100	14.82 (1.76)
School level	Junior high	444	51.6	—
	Senior high	416	48.4	—
Sport type	Ball sports	333	38.7	—
	Track and field	211	24.5	—
	Swimming	132	15.3	—
	Other	184	21.5	—
Exercise experience	<2 years	282	32.8	—
	2–5 years	354	41.2	—
	>5 years	224	26.0	—

The study combined a cross-sectional survey with a short-term longitudinal follow-up. As Figure 1 shows, data collection unfolded in three phases. A pilot survey (*n* = 86) was administered first to check item clarity and scale reliability; item-total correlations below 0.30 prompted the rewording of four items, and all scales reached a Cronbach’s α above 0.80 in the pilot. The formal survey (Time 1) followed 2 weeks later and collected growth mindset, exercise self-efficacy, intrinsic motivation, perceived exercise climate, the demographic variables, and a baseline measure of exercise persistence. One month after Time 1, a follow-up assessment (Time 2) reassessed exercise persistence with the identical instrument (Cook et al., 2002). Of the 912 students surveyed at Time 1, 860 completed the follow-up, an attrition rate of 5.7%; students lost to follow-up did not differ from those retained on gender, school level, or any Time 1 study variable (all *p* > 0.19). All analyses reported below use Time 2 persistence as the outcome and include Time 1 persistence as a covariate, so that the model predicts residual change in persistence rather than its concurrent level. The one-month interval separates predictors from outcome in time, but it does not convert the design into a full panel study, since the mediators and the moderator were measured only once.

**Figure 1:**
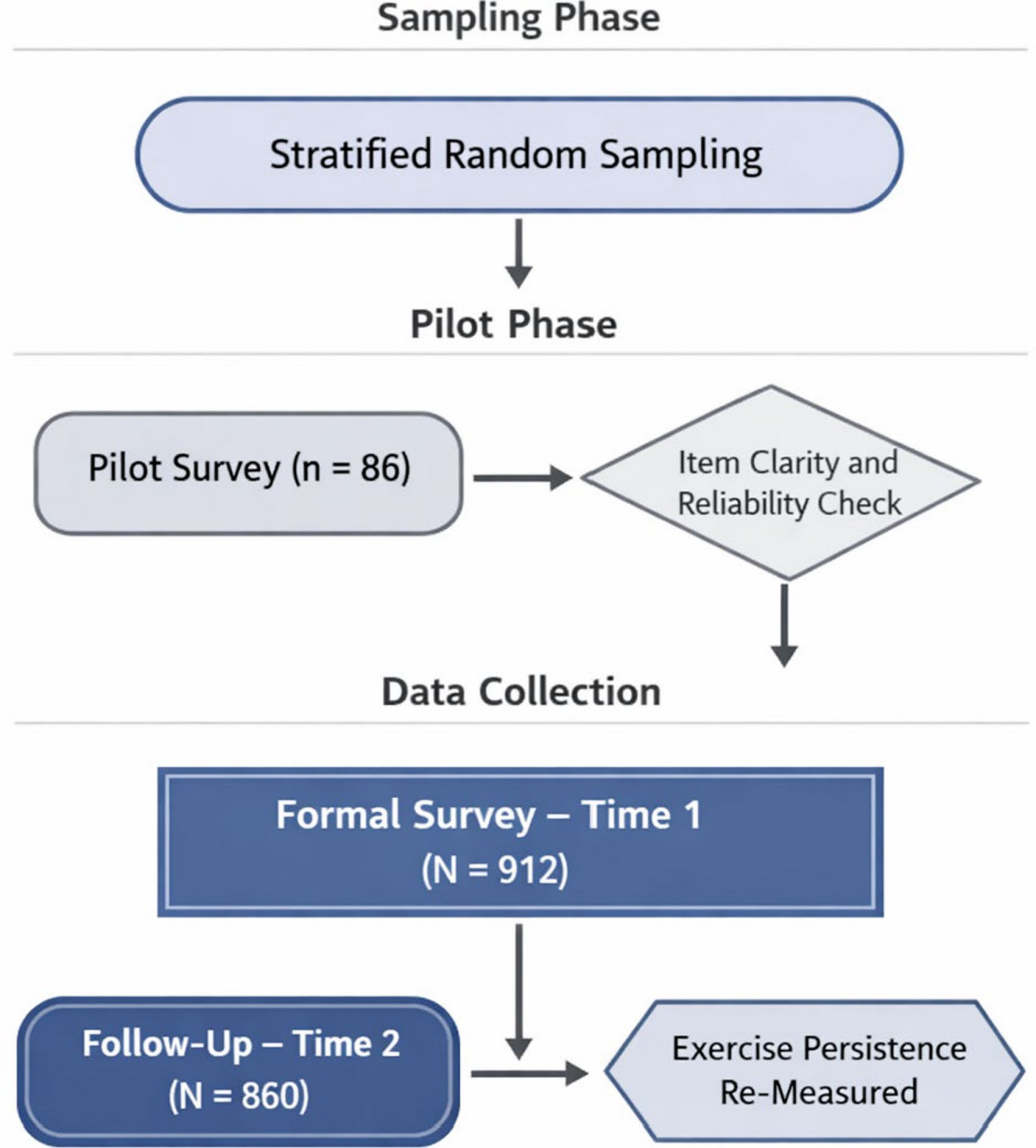
Schematic overview of the research design, illustrating the three-phase data collection process from stratified sampling through pilot testing, formal survey administration (Time 1), and one-month follow-up measurement (Time 2).

The study protocol received approval from the Institutional Review Board of the first author’s university prior to data collection. Because all participants were minors or included minors, written informed consent was obtained from both students and their legal guardians (American Psychological Association, 2002). Participation was voluntary, anonymity was guaranteed, and respondents could withdraw at any stage without consequence.

### 3.2 Instruments and variable measurement

Five constructs were measured with established scales, each adapted into Chinese through a standard translation and back-translation procedure involving two bilingual sport psychology researchers and one independent translator (Brislin, 1970). Discrepancies between the original and back-translated versions were resolved in panel discussion, after which five sport psychology academics and three physical education teachers rated every item for clarity and cultural appropriateness; the item-level content validity index ranged from 0.83 to 1.00 and the scale-level index was 0.92. Cognitive interviews with 15 adolescents then confirmed comprehensibility and led to minor rewording of idiomatic expressions. Several procedural safeguards against common method variance were built into administration: participation was anonymous and voluntary, scales were presented in counterbalanced blocks with item order randomised within blocks, response formats differed across constructs, negatively worded items were interspersed, two attention-check items were embedded, and the outcome was separated from the predictors by a one-month interval (Podsakoff et al., 2003).

Growth mindset was assessed with a revised 6-item Implicit Theories of Athletic Ability Scale on a 6-point Likert format, with higher scores reflecting stronger incremental beliefs (Slater et al., 2012). Exercise self-efficacy was measured with the 10-item Exercise Self-Efficacy Scale, on which respondents rate their confidence in maintaining exercise under various barriers along a 4-point scale (McAuley, 1992). Intrinsic motivation was captured by the 4-item intrinsic motivation subscale of the Sport Motivation Scale, rated on a 7-point continuum; the scale originates in the work of Pelletier et al. (1995), and the parallel academic instrument by Vallerand et al. (1992) informed the wording of the Chinese adaptation. Exercise persistence was operationalized through a 12-item Exercise Persistence Questionnaire spanning behavioral continuity, barrier resistance, and long-term commitment, scored on a 5-point scale (Sallis et al., 2000). Perceived exercise climate was gauged with the 12-item sport-adapted Perceived Motivational Climate Scale, which distinguishes mastery and performance subscales on a 5-point format (Duda and Nicholls, 1992); consistent with the theoretical argument in Section 2.3, the mastery subscale served as the moderator, with higher scores denoting stronger mastery-oriented perceptions. For the two multidimensional instruments, persistence and climate, the subscales were modeled as indicators of a second-order latent factor rather than averaged into a single score, and the second-order specification fitted no worse than the first-order alternative in either case. Chinese versions of all five instruments have been used in previous adolescent research, with reliabilities between 0.82 and 0.92 reported in earlier validation work (Li et al., 2021).

Reliability and validity were then evaluated on the present sample. Internal consistency was indexed by Cronbach’s α and supplemented by composite reliability, which weights items by their standardized factor loadings and is therefore better suited to latent-variable models; convergent validity was judged by the average variance extracted. Because these are standard indices, we report the values rather than the defining formulas. As Table 2 shows, every scale exceeded the conventional thresholds of 0.70 for α and for composite reliability, and all average variance extracted values surpassed 0.50. Single-instrument confirmatory factor analyses returned acceptable fit throughout (CFI > 0.90, RMSEA < 0.08), supporting the structural integrity of the adapted measures.

**Table 2:** Reliability and validity indices for all measurement instruments.

Scale	Items	Cronbach’s α	CR	AVE	CFI
Growth mindset (implicit theories)	6	0.87	0.88	0.55	0.96
Exercise self-efficacy	10	0.91	0.92	0.53	0.93
Intrinsic motivation	4	0.89	0.90	0.69	0.98
Exercise persistence	12	0.93	0.93	0.52	0.92
Perceived exercise climate	12	0.86	0.87	0.51	0.91

Two further pieces of psychometric evidence were sought, since reliability alone says little about whether a translated instrument behaves comparably across groups. Multi-group confirmatory factor analyses tested configural, metric, and scalar invariance across gender and across school level, with changes in the comparative fit index below 0.010 taken as evidence that a more constrained model was tenable (Cheung and Rensvold, 2002). Discriminant validity was assessed both by the Fornell-Larcker criterion and by nested model comparisons in which pairs of latent factors were constrained to correlate perfectly. Results of both sets of analyses appear in Section 4.1.

### 3.3 Data analysis strategy

Before hypothesis testing, the data were screened in several steps. Missing values, which affected fewer than 2% of all cells, were handled by full information maximum likelihood. Outliers were flagged with Mahalanobis distance at *p* < 0.001 and inspected case by case; nine multivariate outliers were retained after inspection because none reflected careless responding, and re-estimating the model without them changed no path by more than 0.01. Skewness and kurtosis for all observed variables fell within acceptable bounds, which supports maximum likelihood estimation, although we used its robust variant throughout because the latent interaction analysis is sensitive to departures from multivariate normality (Kline, 2016). Multicollinearity was checked among the predictors of each endogenous variable; variance inflation factors ranged from 1.18 to 1.63 and tolerances from 0.61 to 0.85, comfortably clear of conventional concern. Intraclass correlations at the school level ranged from 0.02 to 0.06 across the study variables, which, with an average of roughly 29 students per class, implies design effects between about 1.6 and 2.7 and justifies the cluster-robust standard errors used in all structural models (McNeish et al., 2017). Figure 2 outlines the analytic sequence that followed.

**Figure 2:**
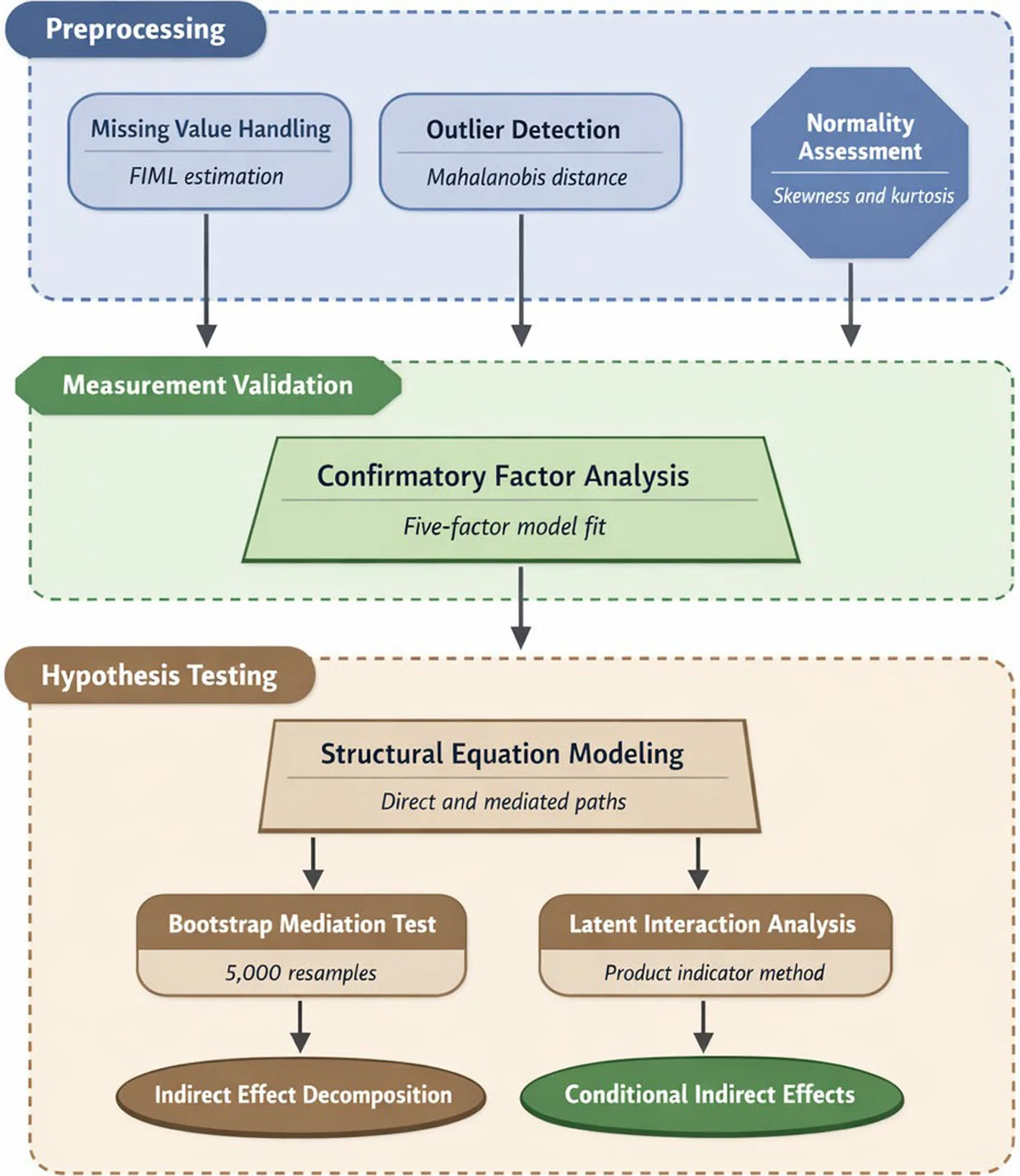
Flowchart of the data analysis strategy, tracing the progression from data preprocessing through confirmatory factor analysis, structural equation modeling, bootstrap-based mediation testing, and latent interaction analysis.

The analysis proceeded in three stages. First, a confirmatory factor analysis with all five latent constructs established the adequacy of the measurement model and its discriminant validity, followed by the invariance tests described in Section 3.2. Model fit was judged against several indices used conventionally in this literature and reported here without their defining formulas: a ratio of *χ*² to degrees of freedom below 3.0, a root mean square error of approximation below 0.08, a standardized root mean square residual below 0.08, and comparative and Tucker-Lewis fit indices at or above 0.90 (Hu and Bentler, 1999).

Second, the structural model was estimated so that direct and mediated paths could be tested simultaneously, with Time 2 persistence as the outcome, Time 1 persistence as a covariate, and school treated as the clustering unit. Mediation was evaluated through bias-corrected bootstrap confidence intervals based on 5,000 resamples, which outperform the Sobel test when the distribution of an indirect effect is non-normal (Hayes, 2022). Both unstandardized and completely standardized solutions are reported, since the former preserves the metric of the instruments and the latter allows comparison with published effect sizes. The total indirect effect decomposes into three specific pathways, one through each mediator alone and one through the sequential chain (Equation 4):

$$ab_{\text{total}}=a_{1}\times b_{1}+a_{2}\times b_{2}+a_{1}\times d_{1}\times b_{2}$$ (4)

Third, the moderating role of perceived exercise climate on the path from growth mindset to self-efficacy was tested with a latent variable interaction. We adopted the unconstrained product indicator method, pairing mean-centred items from the predictor and moderator scales in descending order of their loadings to form six interaction indicators, and estimating the model with robust maximum likelihood (Marsh et al., 2004). Conditional indirect effects were probed one standard deviation above and below the moderator mean, and the index of moderated mediation was examined to determine whether the mediated pathway varied as a function of climate perceptions (Hayes, 2015). Four robustness analyses complete the sequence: adding the demographic variables as covariates, re-estimating the model with cluster-robust standard errors, replacing the concurrent outcome with Time 2 persistence conditional on baseline, and fitting a competing model in which the order of the two mediators is reversed. Their results are reported at the end of Section 4.3.

## 4 Results and analysis

### 4.1 Descriptive statistics and measurement model testing

Table 3 presents means with their 95% confidence intervals, standard deviations, and bivariate correlations among all study variables. Because the scales differ in response format, the means are not directly comparable across constructs and are reported chiefly to document the distribution of each measure. All five constructs correlated positively with one another at *p* < 0.01. The association between growth mindset and exercise persistence was moderate (*r* = 0.41), which is consistent with treating mindset as a distal rather than a proximal predictor. Stronger correlations emerged between the two mediators and the outcome: exercise self-efficacy correlated with persistence at 0.54, and intrinsic motivation at 0.58. The link between self-efficacy and intrinsic motivation (*r* = 0.49) is consistent with the theoretical premise that efficacy feeds into motivational quality, and perceived exercise climate showed modest but consistent associations with every other variable (*r* = 0.28 to 0.37). Latent correlations estimated from the measurement model followed the same pattern and were somewhat larger, as disattenuation would predict, with the highest reaching 0.64 between intrinsic motivation and persistence, still well below the threshold at which discriminant validity would be in doubt.

**Table 3:** Descriptive statistics and correlation matrix for all study variables (*N* = 860).

Variable	M [95% CI]	SD	Skewness	Kurtosis	1	2	3	4	5
1. Growth mindset	4.23 [4.17, 4.29]	0.89	−0.34	−0.18	—				
2. Exercise self-efficacy	2.78 [2.74, 2.82]	0.62	−0.21	0.15	0.43**	—			
3. Intrinsic motivation	4.86 [4.78, 4.94]	1.24	−0.47	−0.09	0.39**	0.49**	—		
4. Exercise persistence	3.52 [3.47, 3.57]	0.78	−0.28	0.22	0.41**	0.54**	0.58**	—	
5. Perceived exercise climate	3.61 [3.56, 3.66]	0.71	−0.15	−0.31	0.34**	0.37**	0.32**	0.28**	—

**p < 0.01.

Before comparing groups, we verified that the measurement model behaved equivalently across them. Multi-group confirmatory factor analysis supported configural invariance across gender (CFI = 0.930, RMSEA = 0.041), and imposing metric and then scalar constraints produced negligible deterioration (∆CFI = − 0.003 and −0.004 respectively, both within the 0.010 criterion), so strong invariance was tenable and observed mean comparisons are interpretable (Cheung and Rensvold, 2002). The same sequence across school level yielded changes no larger than 0.005. Gender differences then emerged on several variables. As Figure 3 illustrates, males scored higher than females on exercise self-efficacy (*t* = 3.12, *p* < 0.01, *d* = 0.22) and exercise persistence (*t* = 2.87, *p* < 0.01, *d* = 0.20), whereas growth mindset and intrinsic motivation did not differ by gender (both *p* > 0.10). These group comparisons are descriptive rather than hypothesis-driven; we report them because gender and school level are entered as covariates in the robustness analyses, and because the invariance evidence they rest on is itself part of the psychometric case for the adapted instruments.

**Figure 3:**
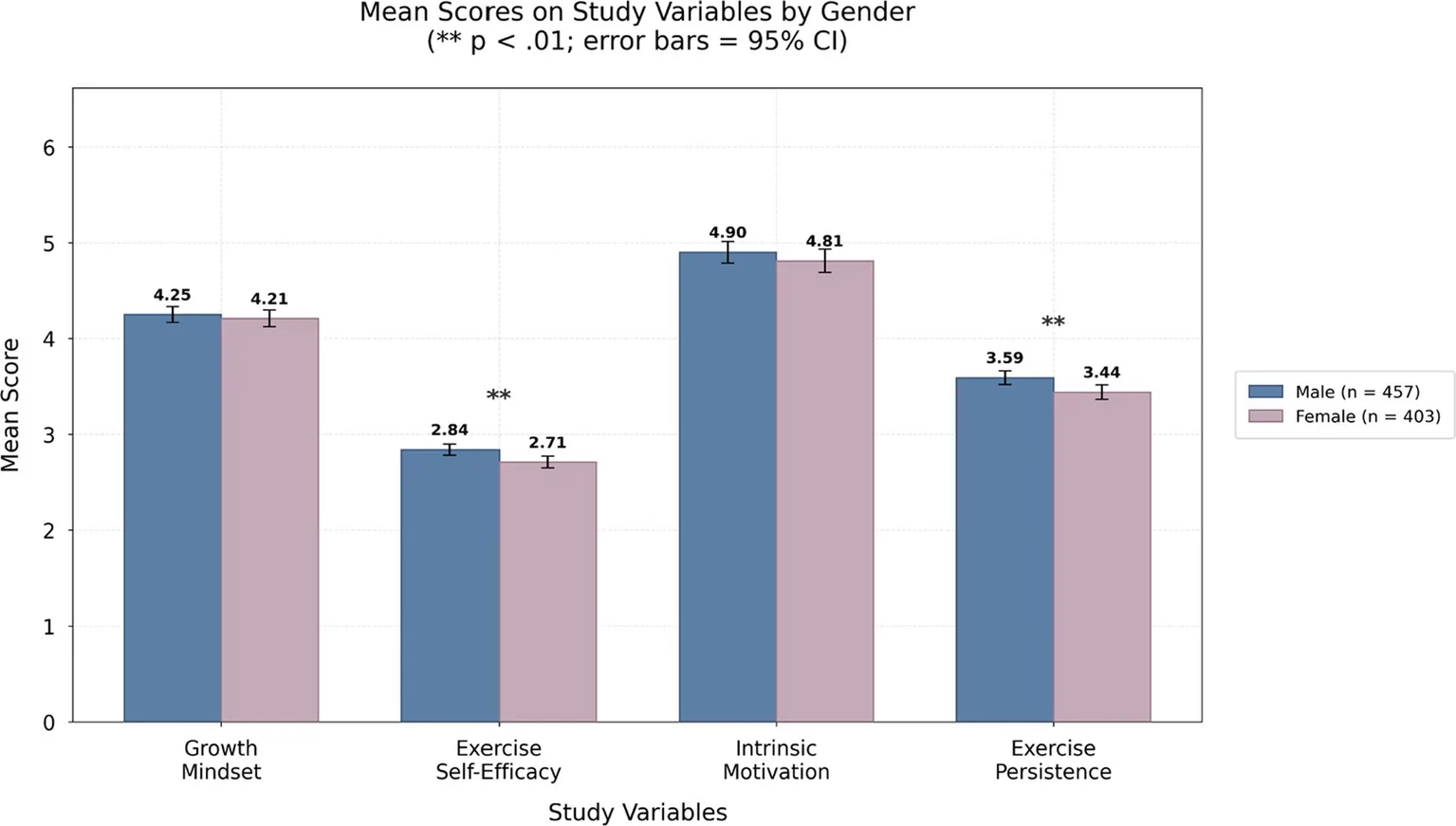
Mean scores on each study variable by gender, with error bars representing 95% confidence intervals; asterisks denote statistically significant group differences at *p* < 0.01.

Sport type produced comparable variation. Figure 4 shows that ball sport participants reported the highest intrinsic motivation (M = 5.14) and persistence (M = 3.71), while track-and-field athletes scored highest on exercise self-efficacy (M = 2.93). One-way analyses of variance confirmed between-group differences for intrinsic motivation, *F*(3, 856) = 4.67, *p* < 0.01, *η*^2^= 0.016, and for persistence, *F*(3, 856) = 3.89, *p* < 0.01, *η*^2^= 0.013; both effects are small by conventional standards (Cohen, 1988). Levene’s test indicated homogeneity of variance in each case (both *p* > 0.21), and Tukey *post hoc* comparisons localised the differences to the contrast between ball sports and swimming (both *p* < 0.05), with no other pairwise contrast reaching significance. Sport type is therefore retained as a covariate in the robustness analyses rather than treated as a substantive moderator.

**Figure 4:**
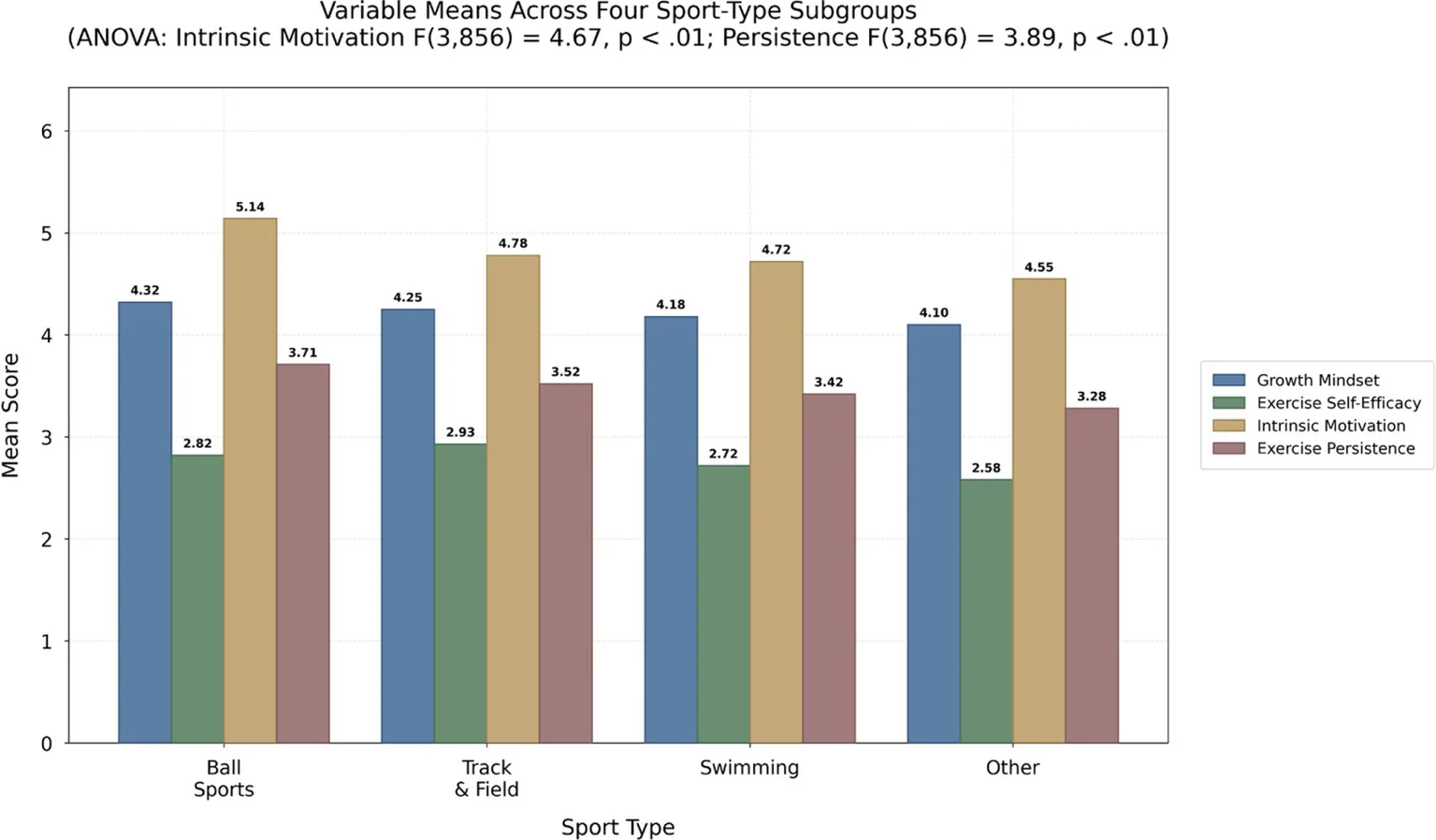
Variable means across four sport-type subgroups (ball sports, track and field, swimming, other), highlighting the pattern of higher intrinsic motivation and persistence among ball sport participants.

The scatter plot in Figure 5 displays the bivariate distribution of growth mindset and exercise persistence scores. It serves two diagnostic purposes: it confirms that the association is approximately linear, which the structural model assumes, and it shows how much dispersion remains around the regression line. That dispersion is itself informative, since a distal predictor whose influence travels through intervening variables should account for a modest share of the variance in the outcome, which is precisely what the mediated model that follows is designed to examine.

**Figure 5:**
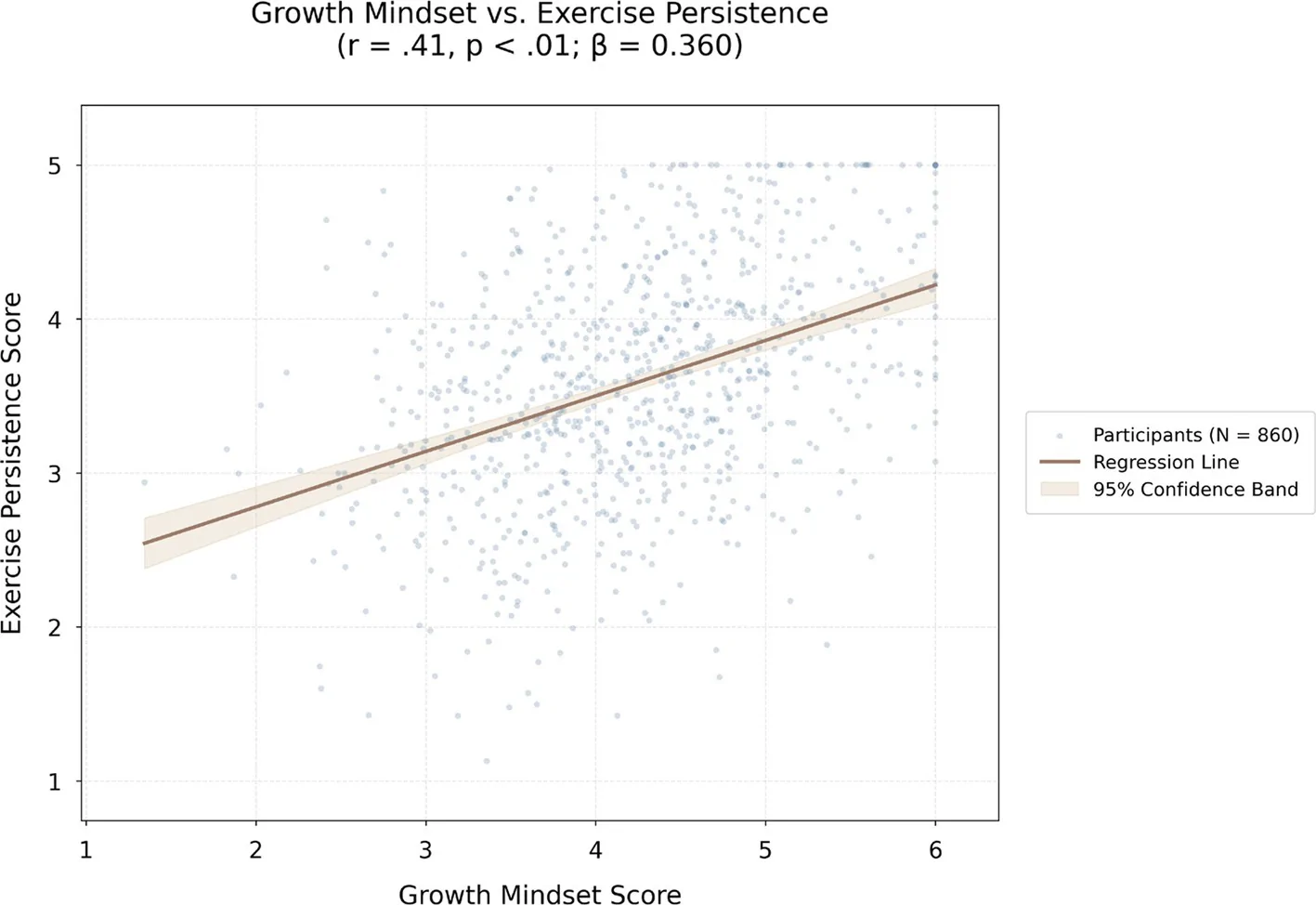
Scatter plot of growth mindset scores (*x*-axis) against exercise persistence scores (y-axis), with the fitted regression line and 95% confidence band overlaid.

Turning to the measurement model, a five-factor CFA was estimated. The model yielded acceptable fit: *χ*²/df = 2.46, CFI = 0.93, TLI = 0.92, RMSEA = 0.041, SRMR = 0.045. All standardized factor loadings exceeded 0.60, and the majority surpassed 0.70. Figure 6 displays these loadings by construct, showing the tightest clustering for intrinsic motivation (range: 0.78–0.88) and the widest spread for perceived exercise climate (range: 0.61–0.82).

**Figure 6:**
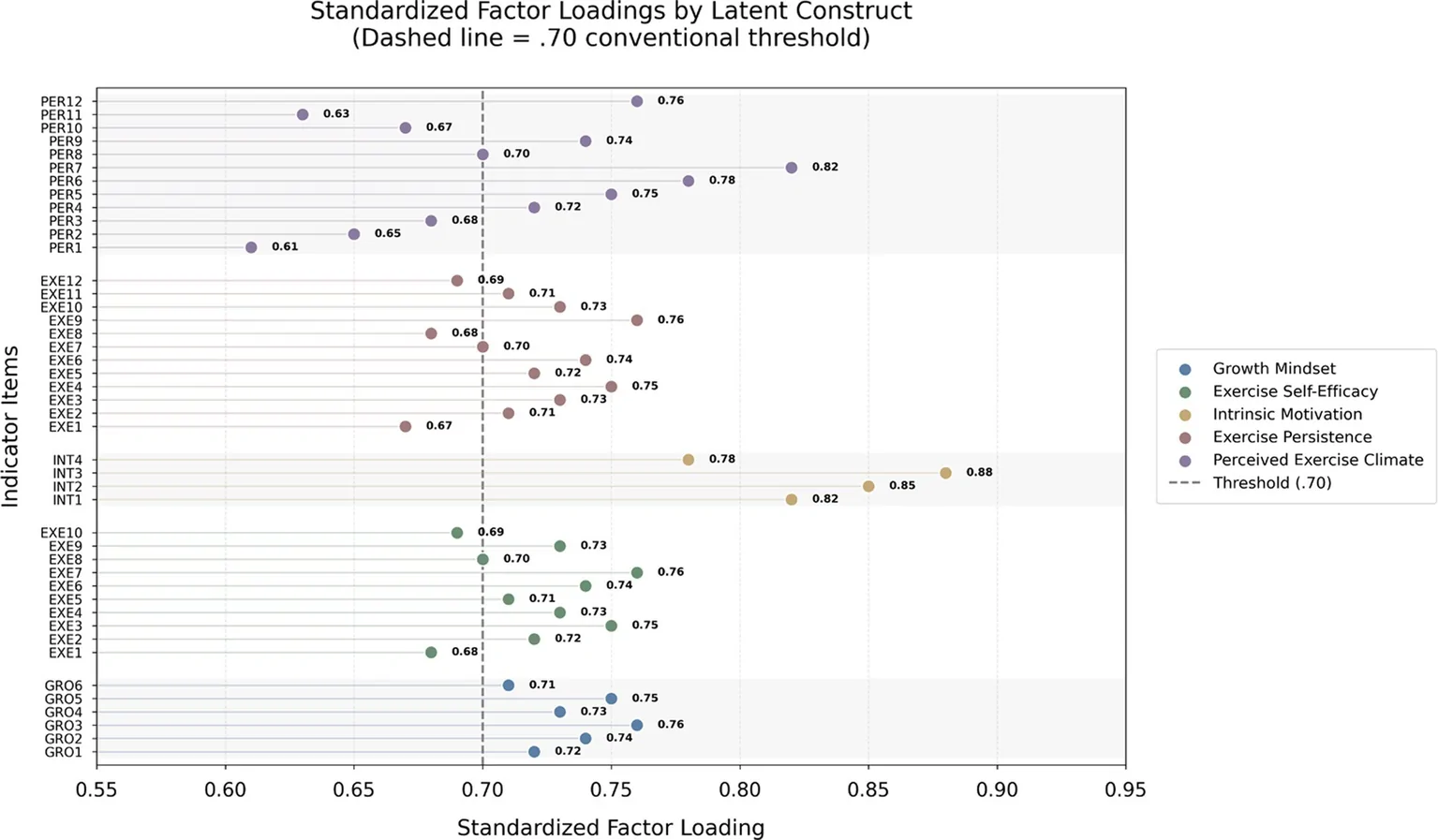
Standardized factor loadings for each indicator organized by latent construct, with the dashed reference line at 0.70 marking the conventional threshold for strong loading.

Discriminant validity was assessed by comparing the average variance extracted for each construct with its squared correlations with the remaining constructs. The square root of each construct’s average variance extracted exceeded its correlations with all other constructs, satisfying the Fornell and Larcker (1981) criterion. Heterotrait-monotrait ratios ranged from 0.38 to 0.72, all below the 0.85 benchmark. Nested model comparisons pointed the same way: constraining any pair of latent factors to correlate at unity produced a significant deterioration in fit in every case, with $\Delta -\chi^{2}$ exceeding 45.3 for a single degree of freedom, *p* < 0.001.

Common method bias was addressed procedurally and statistically. The procedural safeguards described in Section 3.2, anonymity, block counterbalancing, mixed response formats, attention checks, and temporal separation of the outcome, were implemented at the design stage, which is where method variance is most effectively contained (Podsakoff et al., 2003). Statistically, Harman’s single-factor test loaded all items onto one factor accounting for 23.4% of total variance, well below the 40% threshold that would signal concern; we treat this as weak supporting evidence only, since the test is widely regarded as insensitive. A confirmatory factor analytic marker approach was therefore added, using a theoretically unrelated 3-item scale as the marker and comparing baseline, method-C, method-U, and method-R models (Williams et al., 2010). Adding the marker factor did not improve fit appreciably, no path in the structural model shifted by more than 0.02, and every substantive relationship retained its significance. A single unmeasured latent method factor produced the same conclusion. None of these procedures can rule method variance out entirely, a limitation we return to in Section 5.

### 4.2 Structural equation model and mediation testing

The structural model fitted the data well: *χ*²/df = 2.53, CFI = 0.92, TLI = 0.91, RMSEA = 0.043, SRMR = 0.048. Figure 7 presents the standardized path coefficients. Growth mindset predicted exercise self-efficacy (𝑎_1_ = 0.38, B = 0.26, SE = 0.03, *p* < 0.001) and intrinsic motivation (𝑎_2_ = 0.21, B = 0.29, SE = 0.05, *p* < 0.001). Self-efficacy in turn predicted both intrinsic motivation (*d*_1_ = 0.36, B = 0.72, SE = 0.08, *p* < 0.001) and exercise persistence (*b*_1_ = 0.29, B = 0.36, SE = 0.05, *p* < 0.001). Intrinsic motivation exerted the strongest association with persistence (*b*_2_ = 0.39, B = 0.25, SE = 0.03, *p* < 0.001). The residual direct path from growth mindset to persistence remained positive but attenuated (*c*′ = 0.14, B = 0.12, SE = 0.03, *p* < 0.01), a pattern conventionally described as partial mediation. H1 was thus supported. Because the model is estimated on data in which the predictors and mediators share a measurement occasion, the label describes the arrangement of associations rather than a demonstrated sequence of causes.

**Figure 7:**
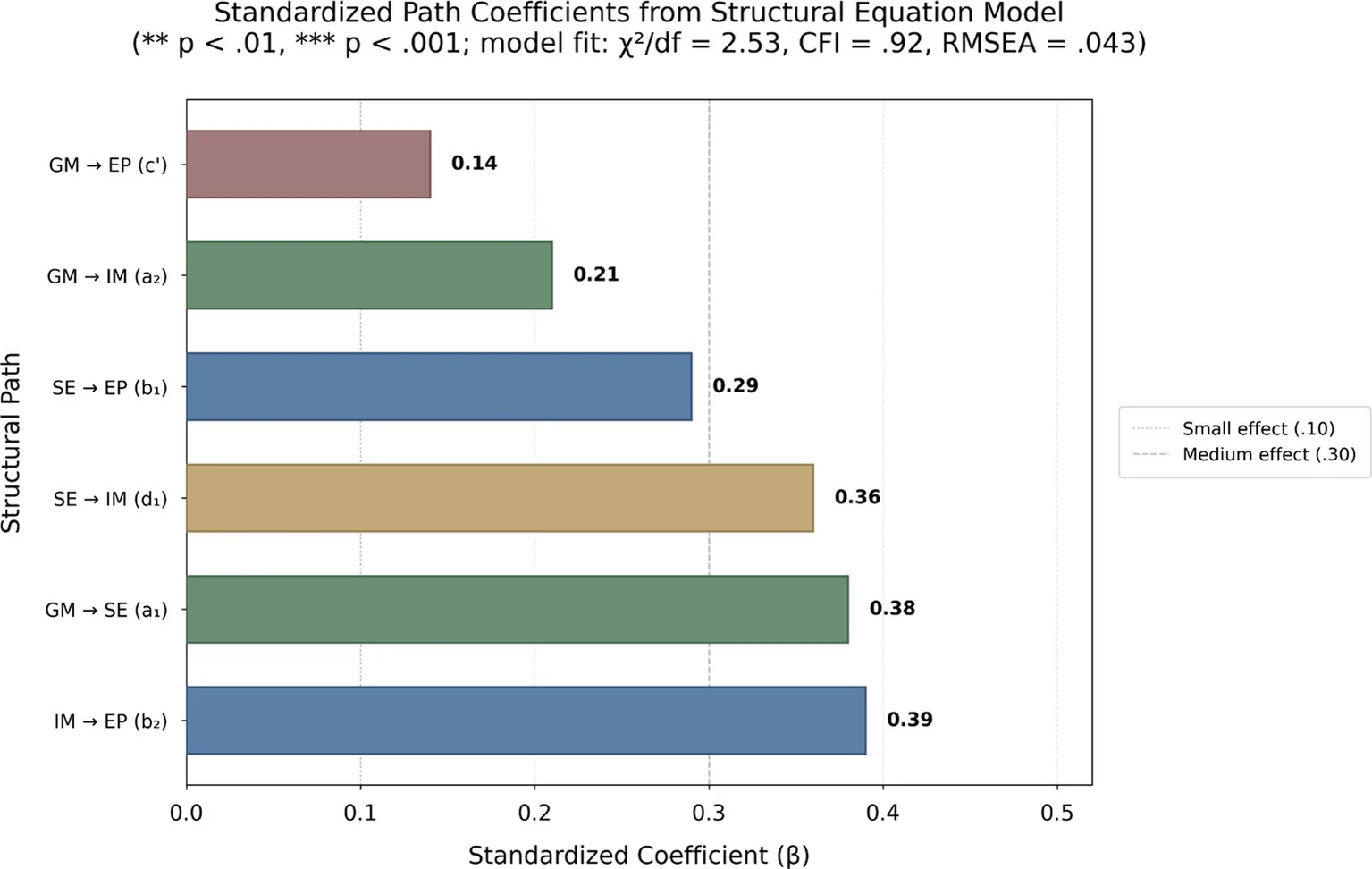
Standardized path coefficients for the structural equation model, with solid lines representing statistically significant paths (*p* < 0.01) and dashed lines indicating the direct effect of growth mindset on exercise persistence after accounting for mediators.

The total effect of growth mindset on persistence decomposes as Equation 5 (standard decomposition formula):

$$c=c^{\prime }+a_{1}b_{1}+a_{2}b_{2}+a_{1}d_{1}b_{2}$$ (5)

Three specific indirect pathways were tested via bias-corrected bootstrap with 5,000 resamples (Hayes, 2022). The first ran through self-efficacy alone (Equation 6):

$$IE_{1}=a_{1}\times b_{1}=.38\times .29=.110$$ (6)

The second passed through intrinsic motivation alone (Equation 7):

$$IE_{2}=a_{2}\times b_{2}=.21\times .39=.082$$ (7)

The third—the chain mediation route—traveled sequentially through both mediators (Equation 8):

$$IE_{3}=a_{1}\times d_{1}\times b_{2}=.38\times .36\times .39=.053$$ (8)

Table 4 summarizes the bootstrap results, reporting each effect in both its unstandardized and its completely standardized form. All three indirect effects produced 95% confidence intervals excluding zero, which supports H2, H3, and H4. The total indirect effect was 0.245 in standardized units, accounting for 63.6% of the total effect (*c* = 0.385).

**Table 4:** Bootstrap results for indirect effects (5,000 resamples), reported in unstandardized and standardized form.

Pathway	B (unstd.)	*β* (std.)	SE	95% CI Lower	95% CI Upper	% of Total
Total indirect	0.196	0.245	0.031	0.187	0.308	63.6
X → M_1_ → Y	0.088	0.110	0.019	0.076	0.150	28.6
X → M_2_ → Y	0.066	0.082	0.017	0.052	0.118	21.3
X → M_1_ → M_2_ → Y	0.042	0.053	0.012	0.033	0.079	13.8
Direct effect (*c*′)	0.112	0.140	0.038	0.067	0.215	36.4

Comparing the three indirect routes reveals a clear hierarchy. The self-efficacy path (IE_1_ = 0.110) carried the largest share of the mediated effect, followed by the intrinsic motivation path (IE_2_ = 0.082), with the chain route (IE_3_ = 0.053) contributing a smaller but non-trivial portion. Pairwise contrasts showed that IE_1_ exceeded ${\mathrm{IE}}_{3}$ (∆ = 0.057, 95% CI [0.021, 0.098]) but did not differ reliably from IE_2_ (∆ = 0.028, 95% CI [−0.009, 0.067]) (Coutts et al., 2023). The chain pathway, though the weakest individual route, is theoretically the most informative: it indicates that self-efficacy and intrinsic motivation are not merely parallel correlates of persistence but are themselves related in the order the integrated framework of Section 2.3 predicts. Whether that ordering reflects the direction in which influence actually runs is a separate question, and one the competing-model analysis reported below addresses only partially.

### 4.3 Moderation testing and simple slope analysis

To test H5, a latent interaction between growth mindset (X) and perceived exercise climate (W) was introduced as a predictor of exercise self-efficacy. Six product indicators were formed from mean-centred item pairs, matched in descending order of their standardized loadings so that each item contributed once, and the model was estimated with robust maximum likelihood. The moderated equation takes the form (Equation 11):

$$M_{1}=a_{1}X+a_{3}W+a_{4}\mathrm{XW}+e$$ (9)

Adding the interaction term improved model fit relative to the constrained model without it (∆χ² = 12.74, ∆df = 1, *p* < 0.001). The interaction coefficient was positive and statistically significant (𝑎_4_ = 0.11, *p* < 0.01), which indicates that perceived exercise climate conditions the strength of the mindset-to-efficacy link. The main effect of climate on self-efficacy also held (𝑎_3_ = 0.19, *p* < 0.001), so mastery-oriented perceptions were associated with higher efficacy independently of mindset orientation.

Simple slope analysis decomposed this interaction at one standard deviation above and below the moderator mean (Marsh et al., 2004). The conditional effect of growth mindset on self-efficacy is expressed as Equation 10:

$$\theta_{X\to M_{1}}=a_{1}+a_{4}W$$ (10)

At high climate perception (+1 SD), the slope was 0.49 (*p* < 0.001); at low climate perception (−1 SD), it dropped to 0.27 (*p* < 0.01). Both slopes remained positive—growth mindset predicted self-efficacy regardless of climate—but the effect was nearly twice as strong when adolescents perceived their exercise environment as mastery-oriented. Figure 8 visualizes this pattern.

**Figure 8:**
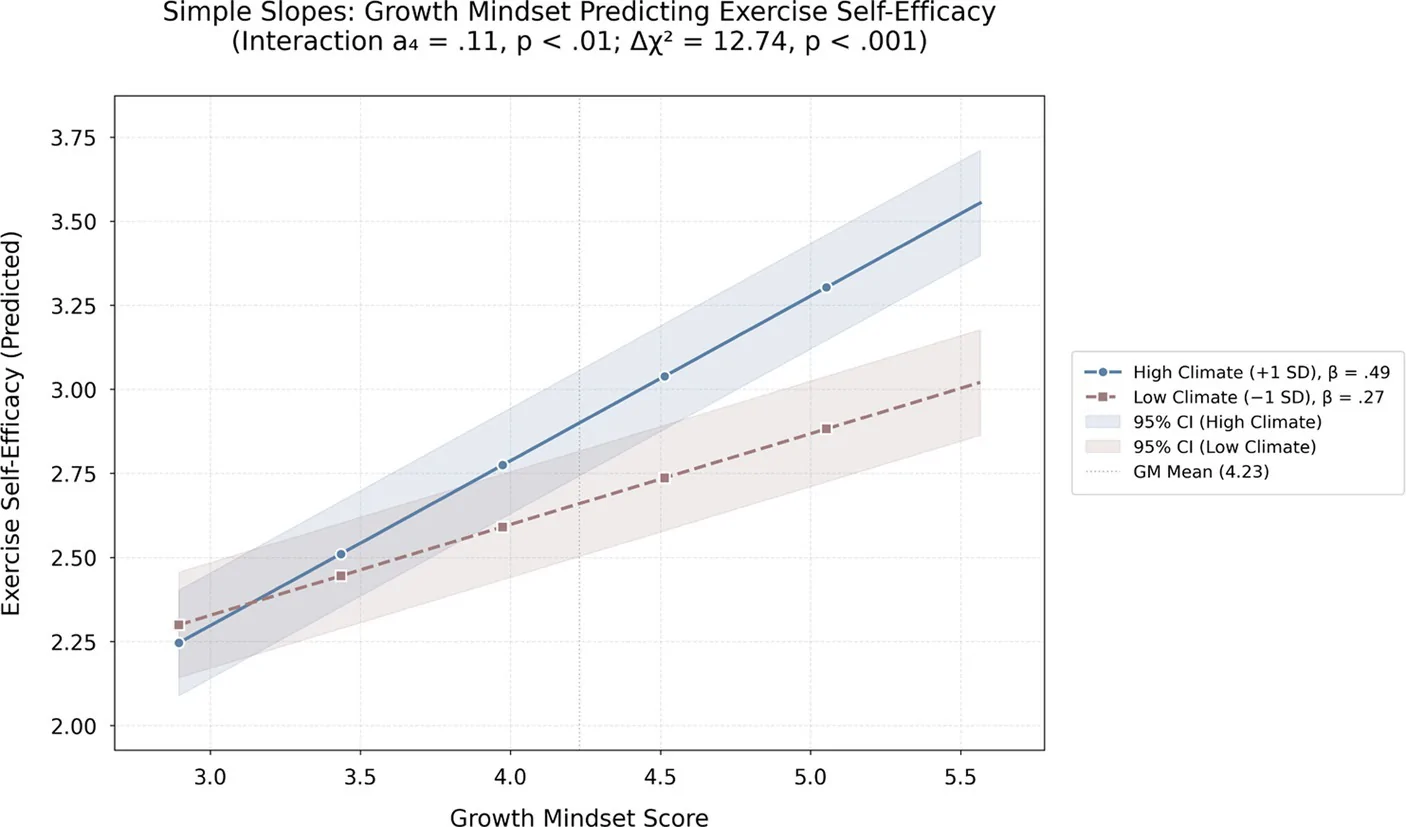
Simple slopes of growth mindset predicting exercise self-efficacy at high (+1 *SD*) and low (−1 *SD*) levels of perceived exercise climate, illustrating the amplifying effect of mastery-oriented climate perceptions on the mindset-efficacy association.

The index of moderated mediation was computed for the indirect path running through self-efficacy to persistence (Hayes, 2015) (Equation 11):

$$\text{Index}=a_{4}\times b_{1}=.11\times .29=.032$$ (11)

Bootstrap analysis (5,000 resamples) produced a 95% CI of [0.011, 0.058], excluding zero and confirming that the strength of the mediated pathway through self-efficacy varies as a function of climate perception. Table 5 presents the conditional indirect effects at both moderator levels alongside the moderated mediation index.

**Table 5:** Moderation and moderated mediation results.

Effect	Estimate	SE	95% CI Lower	95% CI Upper
Interaction (𝑎_4_: X × W → M_1_)	0.110	0.035	0.042	0.178
Conditional indirect at high *W* (+1 *SD*)	0.142	0.024	0.098	0.192
Conditional indirect at low *W* (−1 *SD*)	0.078	0.018	0.046	0.116
Index of moderated mediation	0.032	0.012	0.011	0.058

H5 was supported. The implication is straightforward but consequential: the association between growth mindset and exercise self-efficacy, and by extension its downstream link to persistence, depends in part on whether the surrounding social environment validates effort-based improvement. An adolescent holding strong incremental beliefs but embedded in a climate that prizes innate talent over practice still shows an efficacy advantage, yet the gradient is roughly half that observed among peers in mastery-supportive settings. For intervention design, this pattern suggests that cultivating growth mindset in isolation, without attending to the motivational climate that coaches and peers construct, may yield attenuated returns, a proposition that would need experimental confirmation before it could be relied upon.

Because a moderated serial mediation model rests on a chain of assumptions, we re-estimated it under four alternative specifications and set the results beside the baseline solution. Table 6 lists the key parameters for each specification, and Figure 9 displays the direct and specific indirect effects with their bootstrap intervals so that their stability can be judged at a glance.

**Table 6:** Key parameters under alternative model specifications.

Model specification	𝑎_1_	*b*_1_	*b*_2_	*c*′	Total indirect [95% CI]	CFI / RMSEA
Baseline model	0.38	0.29	0.39	0.14	0.245 [0.187, 0.308]	0.92/0.043
Demographic covariates added	0.37	0.28	0.38	0.13	0.229 [0.174, 0.291]	0.92/0.044
Time 2 persistence, Time 1 controlled	0.37	0.17	0.21	0.07	0.132 [0.089, 0.182]	0.91/0.046
Cluster-robust standard errors	0.38	0.29	0.39	0.14	0.245 [0.169, 0.327]	0.92/0.043
Reversed mediator order	0.21	0.29	—	0.15	0.238 [0.178, 0.305]	0.90/0.051

Coefficients are completely standardized. In the reversed model intrinsic motivation is entered as the first mediator, so 𝑎_1_ denotes the path from growth mindset to intrinsic motivation and the chain parameter is not comparable across rows; ∆BIC = 9.4 favours the hypothesized ordering.

**Figure 9:**
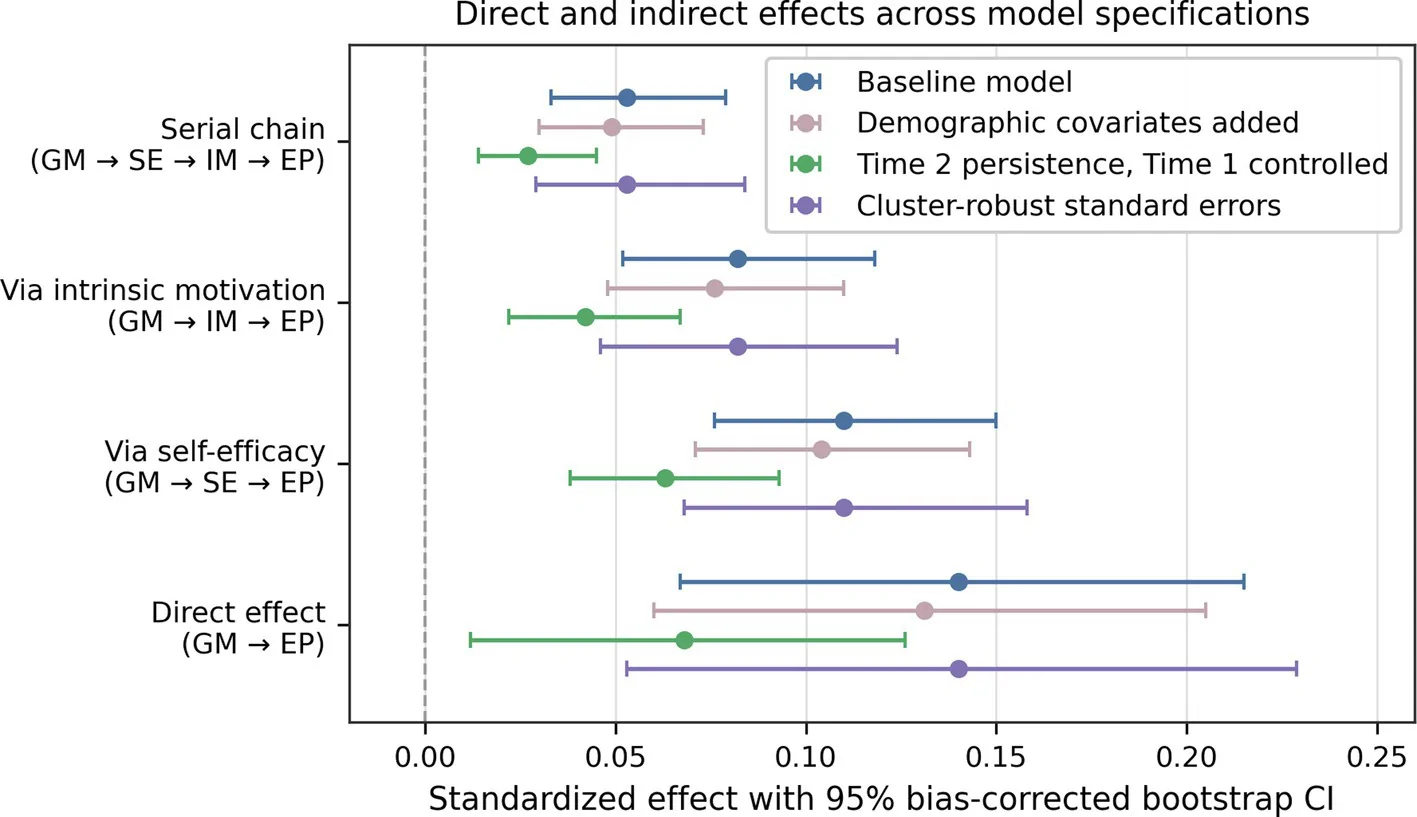
Direct and specific indirect effects with 95% bias-corrected bootstrap confidence intervals under four model specifications: the baseline solution, a model with demographic covariates added, a model predicting Time 2 persistence with Time 1 persistence controlled, and the baseline model with standard errors adjusted for clustering within schools.

Three points emerge. Adding age, gender, school level, exercise experience, and sport type as covariates left every path within 0.02 of its baseline value, so the pattern is not an artefact of demographic composition. Adjusting the standard errors for clustering within the 12 schools widened the confidence intervals by between 18% and 31%, yet all four intervals remained clear of zero. The longitudinal specification, in which Time 2 persistence is modeled with Time 1 persistence controlled and an autoregressive path of 0.58, roughly halved every effect, with the serial indirect effect falling from 0.053 to 0.027 while retaining significance; predicting residual change is a considerably more demanding test than predicting a concurrent level, and attenuation of this magnitude is what one would expect rather than a sign of fragility. The competing model in which intrinsic motivation precedes self-efficacy fitted less well, though not dramatically so, which lends modest support to the ordering we proposed without settling the matter.

Finally, we revisited the question of statistical power with the parameters now in hand. Monte Carlo simulation with 5,000 replications per sample size, seeded with the observed estimates, indicated that 202 cases would suffice for 0.80 power on the self-efficacy path, 291 on the intrinsic motivation path, 347 on the serial chain, and 571 on the index of moderated mediation. At the realized sample of 860, power exceeded 0.99 for all three indirect effects and reached 0.93 for the index, as Figure 10 shows. These figures assume simple random sampling; once design effects of 1.6 to 2.7 are taken into account, effective power for the moderated mediation index falls appreciably, which is why we regard H5 as supported in direction rather than precisely quantified.

**Figure 10:**
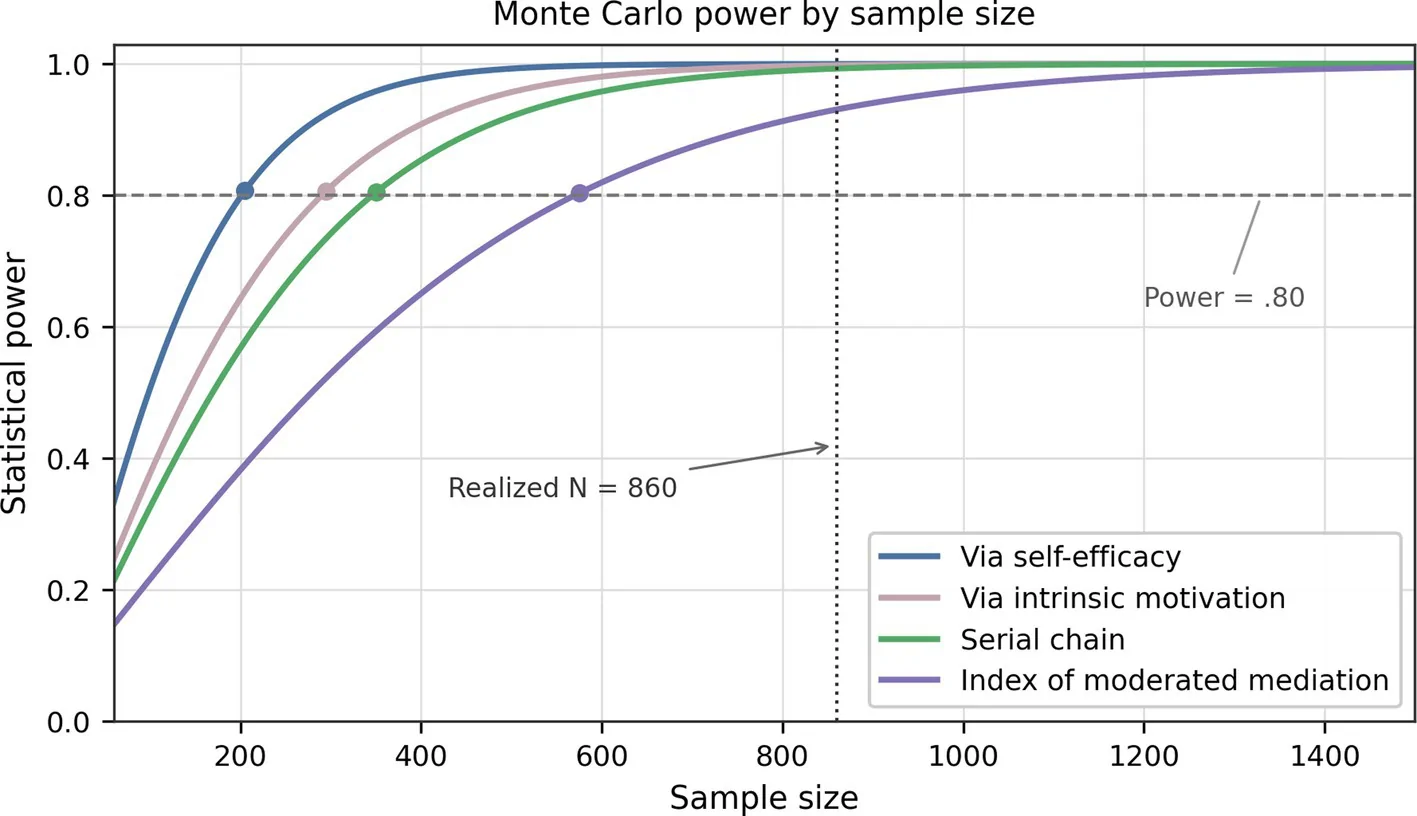
Monte Carlo power curves (5,000 replications per sample size) for the three specific indirect effects and the index of moderated mediation, computed from the observed parameter estimates under an assumption of simple random sampling; markers indicate the smallest sample reaching 0.80 power and the dotted line marks the realized sample of 860.

## 5 Discussion

The findings of this study converge on a central claim: growth mindset is associated with adolescent exercise persistence not merely along a direct cognitive route but primarily through a sequential motivational chain, and that chain is sensitive to the social environment in which it operates. Each element of the picture warrants separate consideration, and we begin by setting out how far each hypothesis was borne out.

Taken hypothesis by hypothesis, H1 received support: growth mindset predicted persistence before mediators entered the model (c = 0.385) and retained a smaller but reliable residual association afterwards (*c*′ = 0.14). H2 and H3 were likewise supported, with self-efficacy and intrinsic motivation each carrying a significant indirect effect, and the confidence intervals for both excluded zero under every specification we examined. H4, the chain hypothesis, was supported as well, though the effect was the smallest of the three and shrank furthest once baseline persistence was controlled, which counsels against overstating it. H5 was supported, with the index of moderated mediation excluding zero, but it is also the estimate for which our sample provides the least power, and the conditional effects at high and low climate should be read as an indication of direction more than as a precise quantification of magnitude.

That growth mindset showed a direct association with exercise persistence (*c*′ = 0.14) accords with what Dweck’s framework would anticipate, since believing one’s athletic abilities are malleable should orient an adolescent toward effort-sustained engagement. The modest size of this residual effect once mediators were introduced is nonetheless instructive. The belief by itself does not appear to be the main engine of persistence; it seems rather to set cognitive preconditions under which more proximal motivational processes operate. This pattern mirrors observations from academic contexts, where mindset effects on achievement tend to shrink once mediating variables such as goal orientation and self-regulation are taken into account, and it fits the broader picture emerging from recent meta-analytic work, in which average mindset effects are small and highly conditional (Burnette et al., 2023; Macnamara and Burgoyne, 2023). The exercise domain, it appears, follows a similar logic.

The mediation results fill in the how and the why. Self-efficacy carried the largest single indirect effect (IE_1_ = 0.110), which is intuitive: an adolescent who believes athletic skill is developable is likely, almost by definition, to hold higher confidence in her own potential for improvement. Less obvious, and this is what the chain pathway (IE_3_ = 0.053) contributes, is that the efficacy advantage does not simply push persistence along on its own. It is also associated with stronger intrinsic motivation, in the manner that self-determination theory predicts when the need for competence is met. It is worth distinguishing here between what replicates and what extends. That efficacy and autonomous motivation each predict sustained physical activity is well established and our estimates fall squarely within the ranges reported meta-analytically (Dishman et al., 2004; Vasconcellos et al., 2020; Ntoumanis et al., 2021); the same is true of the bivariate mindset-persistence link (Kasimatis et al., 1996; Stenling et al., 2022). What has not previously been estimated in the exercise domain is the arrangement of these variables as one sequence, and the finding that the sequential route survives alongside the two parallel routes is the study’s principal empirical addition. Its size, however, should temper enthusiasm: the chain accounted for roughly a seventh of the total association, and less once baseline persistence was controlled.

From a theoretical standpoint, this architecture offers a workable integration of two traditions that have often operated in isolation. Social cognitive theory has emphasized self-efficacy as the primary regulator of behavioral persistence, with comparatively little attention to the quality of motivation that sustains effort over time. Self-determination theory has concentrated on motivational regulation and need satisfaction without fully specifying the upstream cognitive conditions that determine whether needs are met in the first place. Our model connects them by positioning growth mindset as the distal cognitive input, self-efficacy as the appraisal through which that input enters the motivational system, and intrinsic motivation as the proximal energizer of sustained action. The result is less a competition between theories than a division of labour, with social cognitive theory accounting for how beliefs become expectations and self-determination theory for how expectations become motives. We would add one caution: the competing model in which the mediators are reversed fitted only modestly worse, so the division of labour we describe is the better-supported arrangement rather than the only defensible one.

The moderation findings add a layer of ecological realism to this individual-level account. Perceived exercise climate conditioned the first link in the chain, so that the mindset-efficacy gradient was close to twice as steep under high mastery climate perceptions as under low. The implication is somewhat sobering. Even a strongly growth-minded adolescent appears to convert those beliefs into efficacy less readily where coaches emphasize talent selection over skill development, where peers mock effort rather than celebrate improvement, or where the athletic culture of a school treats physical ability as something one either has or lacks. The belief looks necessary but not sufficient; the environment has to ratify it. This may be especially pertinent in the Chinese educational context, where competitive ranking and examination pressure can generate performance-oriented climates even inside physical education classes nominally designed for enjoyment and health, a possibility consistent with the climate literature more broadly (Ntoumanis and Biddle, 1999; Ames, 1992) though not directly tested here.

Several practical implications follow, subject to an important caveat: the evidence is correlational, and an association observed in survey data does not establish that changing one variable will change another. What follows should therefore be read as hypotheses for intervention research rather than as prescriptions. First, school-based programmes aiming to promote long-term adherence might treat growth mindset as an entry point rather than a standalone strategy. Brief mindset work, such as teaching students that athletic skills respond to deliberate practice, may initiate a cascade through efficacy and motivation, but the recent trial literature suggests such effects are modest and highly dependent on context (Macnamara and Burgoyne, 2023; Yeager et al., 2019). Second, physical education teachers and coaches could be trained to structure mastery-oriented climates by emphasizing personal improvement over normative comparison, rewarding effort and strategy rather than innate talent, and framing setbacks as diagnostic information rather than verdicts on ability. Third, the differences across sport types were small and exploratory, but the possibility that team-based formats generate mastery cues naturally would be worth testing directly rather than assuming.

Several boundary conditions deserve acknowledgement. Although exercise persistence was reassessed after 1 month and modeled with baseline persistence controlled, the predictors, mediators, and moderator were all measured on a single occasion, so the mediation results describe hypothesized associations rather than demonstrated causal mechanisms; cross-sectional estimates of longitudinal mediation can be substantially biased even when the underlying process is real (Maxwell and Cole, 2007). Reverse influence remains plausible, since adolescents who persist may gradually develop stronger growth beliefs. Residual confounding is a further concern: unmeasured factors such as physical fitness, family support, timetable pressure, and teacher quality could account for part of the observed pattern. All variables were self-reported, which carries the risk of socially desirable responding and shared method variance, and the statistical checks reported in Section 4.1 reduce but cannot eliminate that possibility. One month is also a short window in which to observe persistence, a construct defined by endurance over much longer horizons. The sample, though geographically diverse within China, may not generalize to settings where attitudes toward effort, talent, and physical activity differ. Future work should employ experimental or multi-wave panel designs, extend the framework to clinical or at-risk populations, and incorporate objective measures of exercise behavior such as accelerometry.

## 6 Conclusion

This study set out to answer a deceptively simple question: how does believing that athletic ability can grow relate to actually sticking with exercise? To address it, we constructed a moderated serial mediation model grounded in social cognitive theory and self-determination theory, surveyed 860 Chinese adolescents across eastern, central, and western regions, and analysed the data through confirmatory factor analysis, invariance testing, structural equation modeling with cluster-robust standard errors, bootstrap-based mediation testing, and latent interaction analysis. The design paired a Time 1 survey with a one-month follow-up measure of persistence, which served as the outcome with baseline persistence controlled, and all five instruments showed satisfactory psychometric properties after cultural adaptation.

The core finding can be stated concisely. Adolescents who regard athletic ability as developable reported greater persistence in exercise, and that association operates predominantly through two sequential mediators, exercise self-efficacy and intrinsic motivation. The chain pathway running from mindset through efficacy through motivation to persistence was confirmed alongside two parallel single-mediator routes, and the three indirect pathways together accounted for roughly two-thirds of the total association, leaving a modest but reliable direct link. Perceived exercise climate moderated the entry point of the chain, with mastery-oriented perceptions almost doubling the strength of the mindset-to-efficacy path. Each of the five hypotheses received support, with the chain effect the smallest and the moderated mediation index the least precisely estimated of them.

The theoretical contribution of this work is twofold. First, the tested chain model offers a concrete specification of how two major motivational theories connect within the exercise domain: social cognitive theory’s emphasis on efficacy beliefs and self-determination theory’s focus on autonomous motivation are not rival explanations so much as adjacent stages of one transmission process, in which mindset is associated with self-efficacy, efficacy with the competence satisfaction that accompanies intrinsic motivation, and intrinsic motivation with sustained engagement. Second, the moderation finding shows that individual-level cognitive orientations do not operate in a social vacuum. How efficiently growth beliefs translate into self-efficacy depends on whether the surrounding climate validates effort-based improvement, a point that prior mindset research in sport had theorized but rarely tested.

For practice, three suggestions emerge, all of them conditional on the causal ordering being confirmed experimentally. Adolescent physical activity programmes might incorporate brief growth mindset components, built around the message that athletic competence responds to deliberate practice, as a cognitive foundation for sustained engagement. Such components are likely to work better when embedded in mastery-oriented climates, which makes training coaches and physical education teachers to emphasize personal progress, reward strategic effort, and reframe failure as information a complement to mindset work rather than an afterthought. The observation that ball sport participants reported higher intrinsic motivation and persistence than peers in individual activities hints that collaborative formats may generate mastery cues naturally, a design principle worth testing rather than adopting on the strength of a small descriptive difference.

Several limitations temper these conclusions and point toward future inquiry. The design measured predictors, mediators, and moderator concurrently, and a one-month follow-up of the outcome, while it establishes temporal precedence for persistence, cannot establish the causal direction of the mediating chain. It remains possible that persistent exercisers gradually adopt growth beliefs as a retrospective account of their own behavior rather than the reverse. Panel studies with three or more waves would permit cross-lagged analysis, and randomised mindset training with downstream tracking of efficacy, motivation, and behavior would provide the strongest evidence. All variables were self-reported, so objective indicators such as accelerometer-derived activity or attendance records would strengthen future designs. The clustered sampling frame was addressed with robust standard errors, but a larger number of schools would permit properly multilevel estimation. The sample was drawn entirely from one cultural context, and Chinese adolescents operate under distinctive pressures, including academic competition, filial expectations, and particular attitudes toward physical talent, that may shape the mindset-persistence relationship differently elsewhere. Our model also examined mastery climate alone; parental sport socialisation, peer exercise norms, and access to facilities may condition the same process and deserve systematic investigation.

Despite these caveats, the study provides early evidence, in a Chinese adolescent sample, that growth mindset is associated with exercise persistence through a theoretically grounded chain of self-efficacy and intrinsic motivation, and that this chain is stronger where the exercise climate is perceived as mastery-oriented. The message for researchers and practitioners alike is that mindset, motivation, and environment are not competing explanations for why adolescents keep exercising; they are interlocking pieces of the same puzzle.

## Data Availability

The datasets generated and analysed for this study are available from the corresponding author on reasonable request.
